# Supplementary material for: Fluctuating light experiments and semi-automated plant phenotyping enabled by self-built growth racks and simple upgrades to the IMAGING-PAM
Source: Plant Methods. 2019 Dec 23;15:156. doi: 10.1186/s13007-019-0546-1 (PMC6927185; doi:10.1186/s13007-019-0546-1)

Additional File 1

A **Extension or single unit Fluctuating Light Shelf**

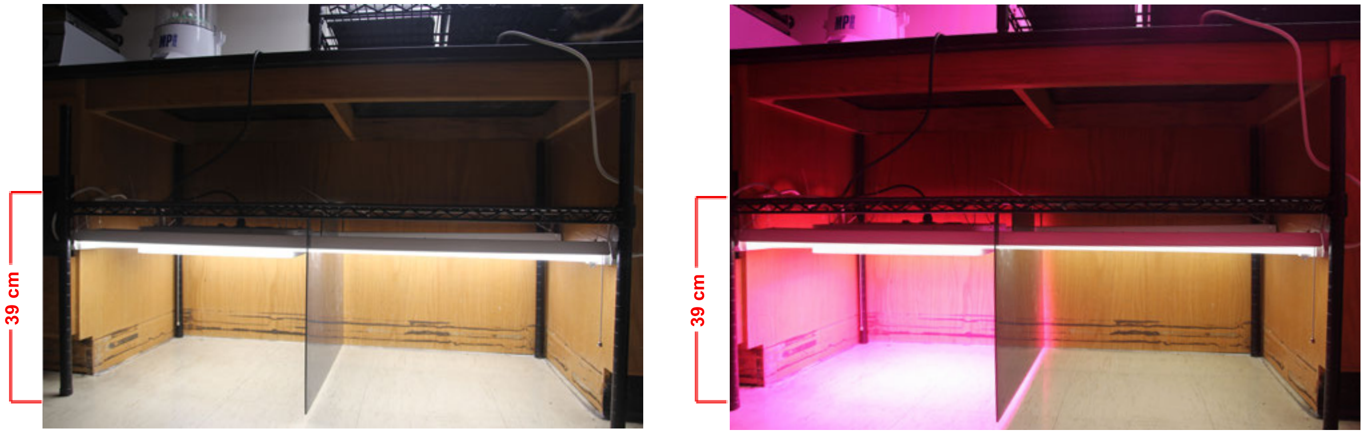

Fluctuating Light **Constant Light** **Fluctuating Light** **Constant Light**

B **Full Fluctuating Light Growth Rack**

Background Light Mode **Fluctuating Light Mode**

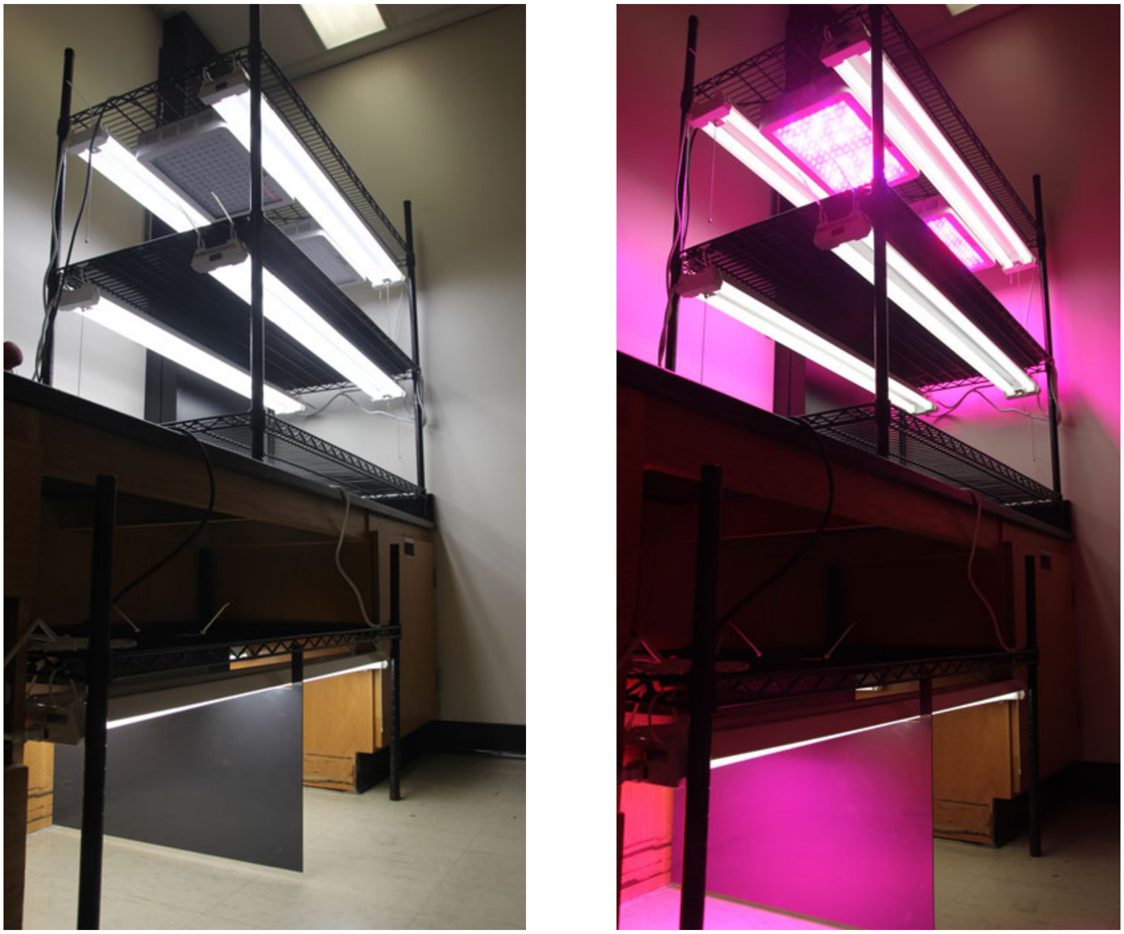

Supplement: Supplementary file 1 — Additional file 1. (A) Background and fluctuating light mode of the growth racks. B) Extension or single unit fluctuating light shelf. Shown are both operation modes. [file 13007_2019_546_MOESM1_ESM.pdf]
