# Supplementary material for: Fluctuating light experiments and semi-automated plant phenotyping enabled by self-built growth racks and simple upgrades to the IMAGING-PAM
Source: Plant Methods. 2019 Dec 23;15:156. doi: 10.1186/s13007-019-0546-1 (PMC6927185; doi:10.1186/s13007-019-0546-1)
Supplement: Supplementary file 3 — Additional file 3. Schematics of the sample holder kit. Sample crate and plant pot holders are compatible with the IMAGING PAM. [file 13007_2019_546_MOESM3_ESM.pdf]

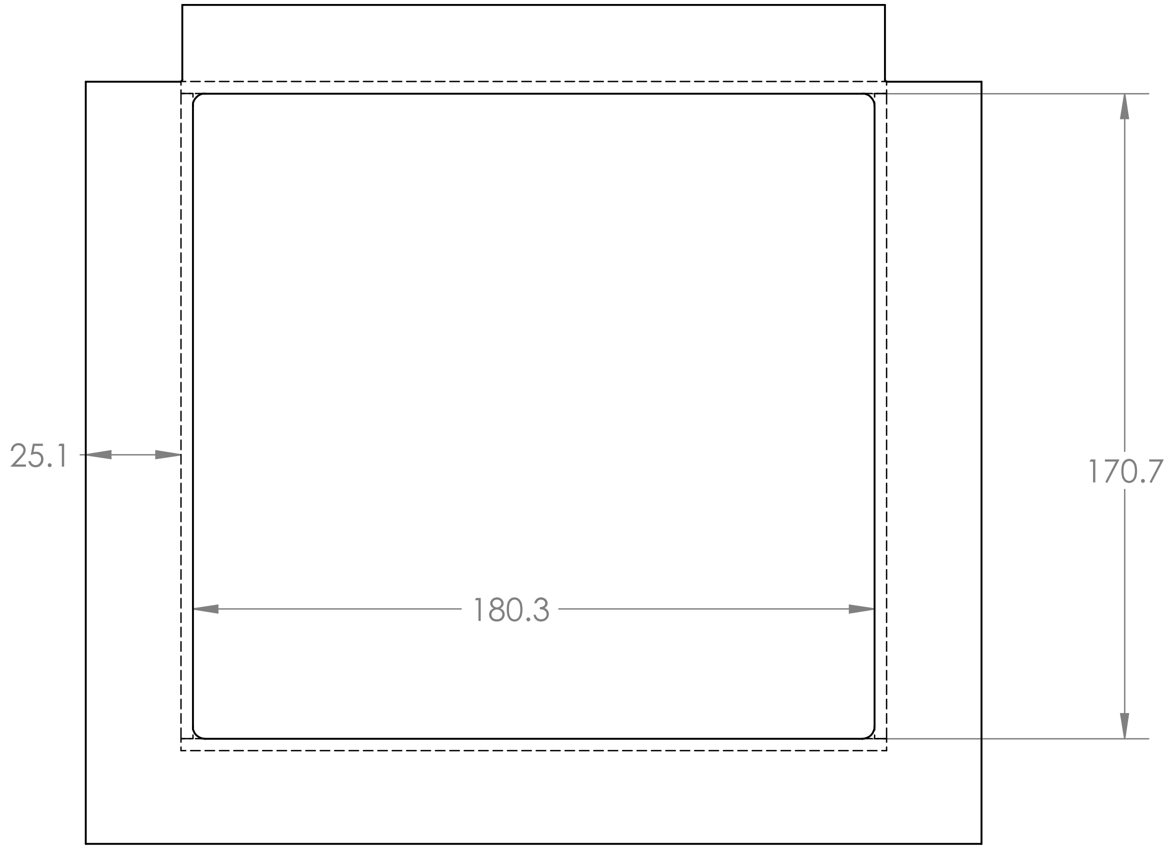

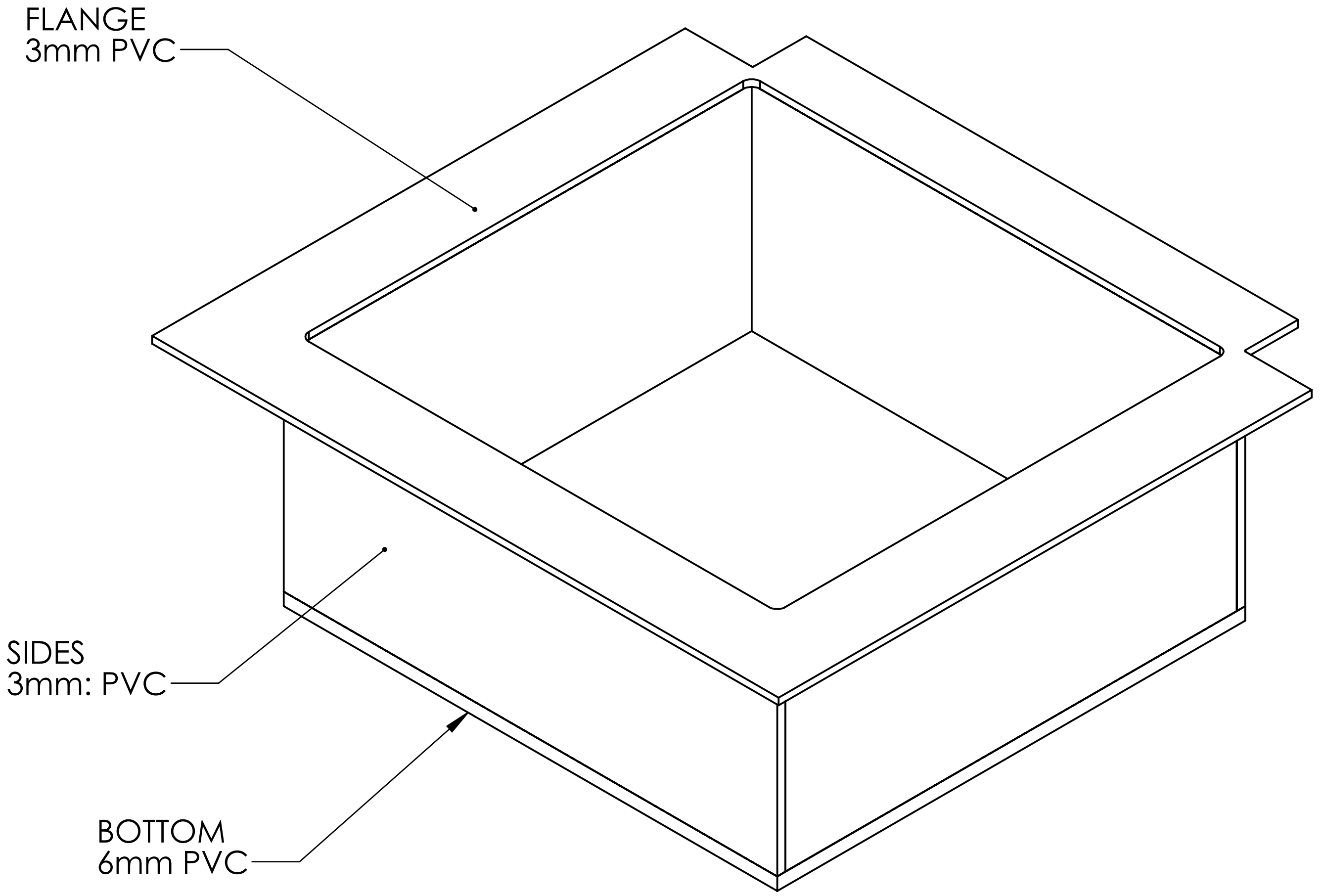

SOLVENT WELDED ASSEMBLY

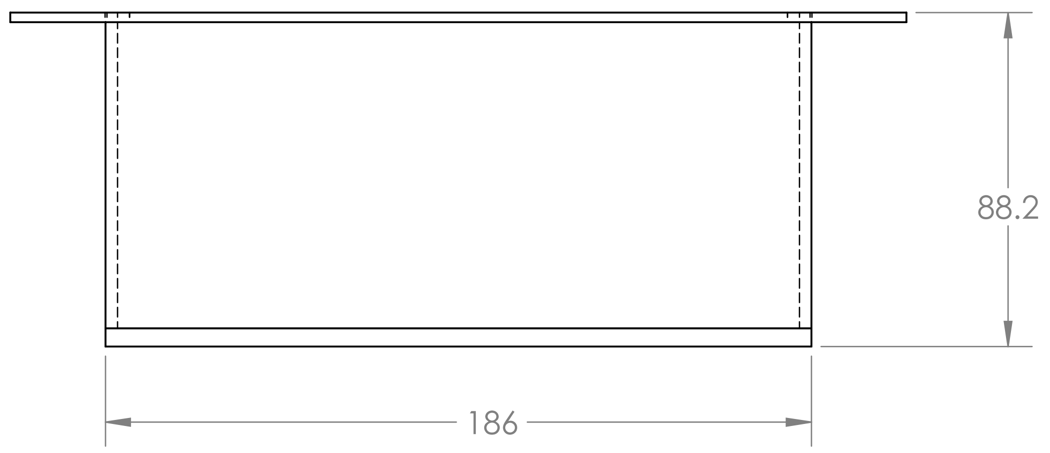

THIS DRAWING IS PROPERTY OF WASHINGTON STATE UNIVERSITY DEPARTMENT OF TECHNICAL SERVICES.
ANY REPRODUCTION OR FABRICATION PROHIBITED WITHOUT WRITTEN PERMISSION OF THE OWNER.

UNLESS OTHERWISE SPECIFIED:		WSU TECHNICAL SERVICES
DIMENSIONS ARE IN MM<br>TOLERANCES:<br>FRACTIONAL ± 1/64<br>ANGULAR: MACH ± .1° BEND ±	DRAWN: SAVAGE<br>DATE: 6/5/19<br>SCALE: 1:2<br>SHEET:<br>JOB #: 430B/19	TITLE:<br>PLANT PHOTO CRATE
MATERIAL: AS NOTED<br>FINISH:	COMMENTS:	SIZE: B · DWG. NO.: · REV:
DO NOT SCALE DRAWING	FILEPATH:	

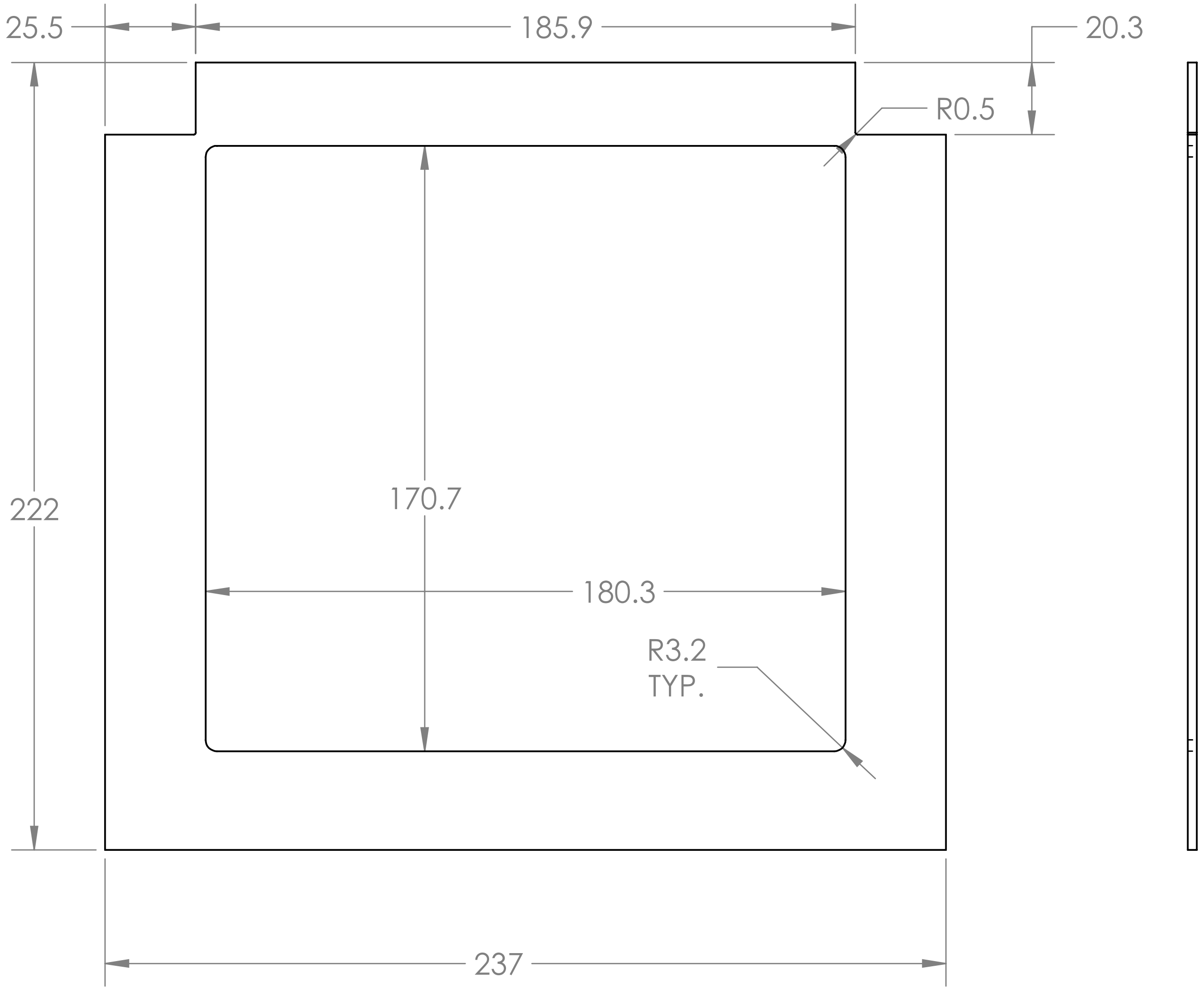

FLANGE:
3MM PVC

UNLESS OTHERWISE SPECIFIED:			WSU TECHNICAL SERVICES
DIMENSIONS ARE IN MM<br>TOLERANCES:<br>FRACTIONAL ± 1/64<br>ANGULAR: MACH ± .1° BEND ±	DRAWN	SAVAGE	TITLE:
	DATE	6/5/19	PLANT PHOTO CRATE
	SCALE	1:2	
	SHEET		
	JOB #	430B/19	
MATERIAL<br>AS NOTED	COMMENTS:		SIZE B | DWG. NO. | REV
FINISH			
DO NOT SCALE DRAWING	FILEPATH:		

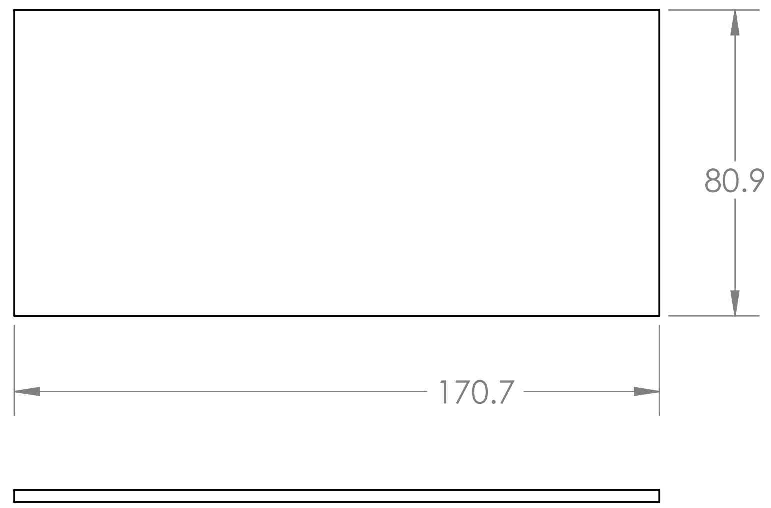

SIDES
QNT'Y: 2
MAT'L: 3MM PVC

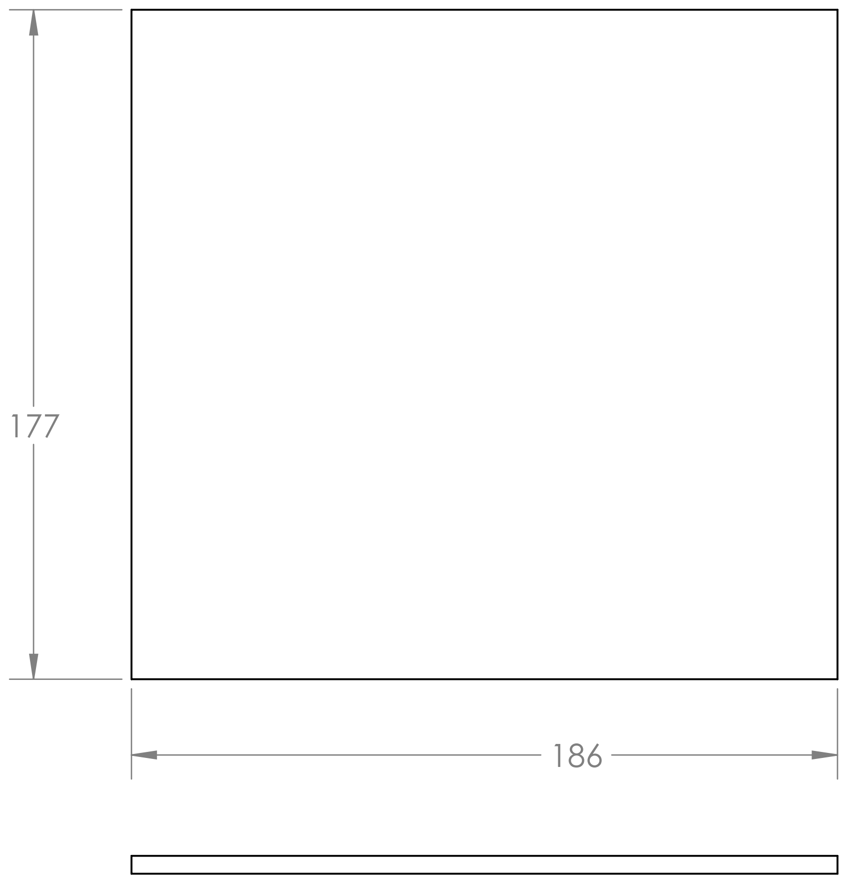

BOTTOM
MAT'L: 6MM PVC

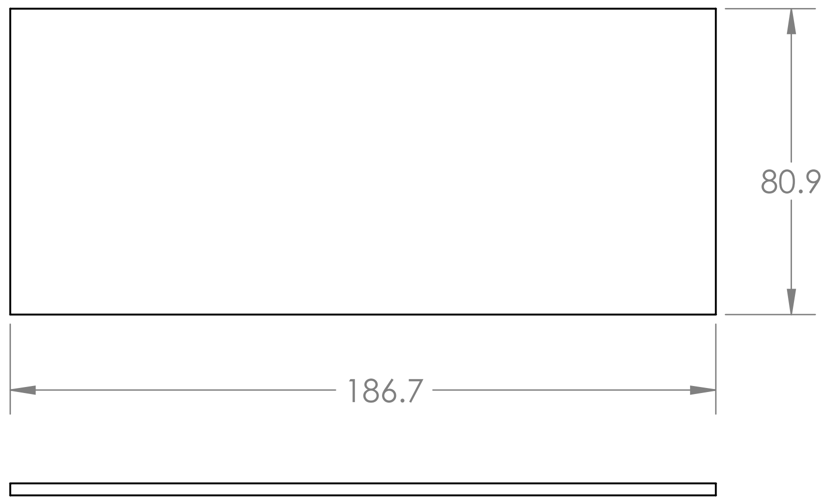

FRONT AND BACK
QNT'Y: 2
MAT'L: 3MM PVC

UNLESS OTHERWISE SPECIFIED:			WSU TECHNICAL SERVICES
DIMENSIONS ARE IN MM<br>TOLERANCES:<br>FRACTIONAL ± 1/64<br>ANGULAR: MACH ± .1° BEND ±	DRAWN	SAVAGE	TITLE:
	DATE	6/5/19	PLANT PHOTO CRATE
	SCALE	1:2	
	SHEET		
	JOB #	430B/19	
MATERIAL<br>AS NOTED	COMMENTS:		SIZE B / DWG. NO. / REV
FINISH			
DO NOT SCALE DRAWING	FILEPATH:		

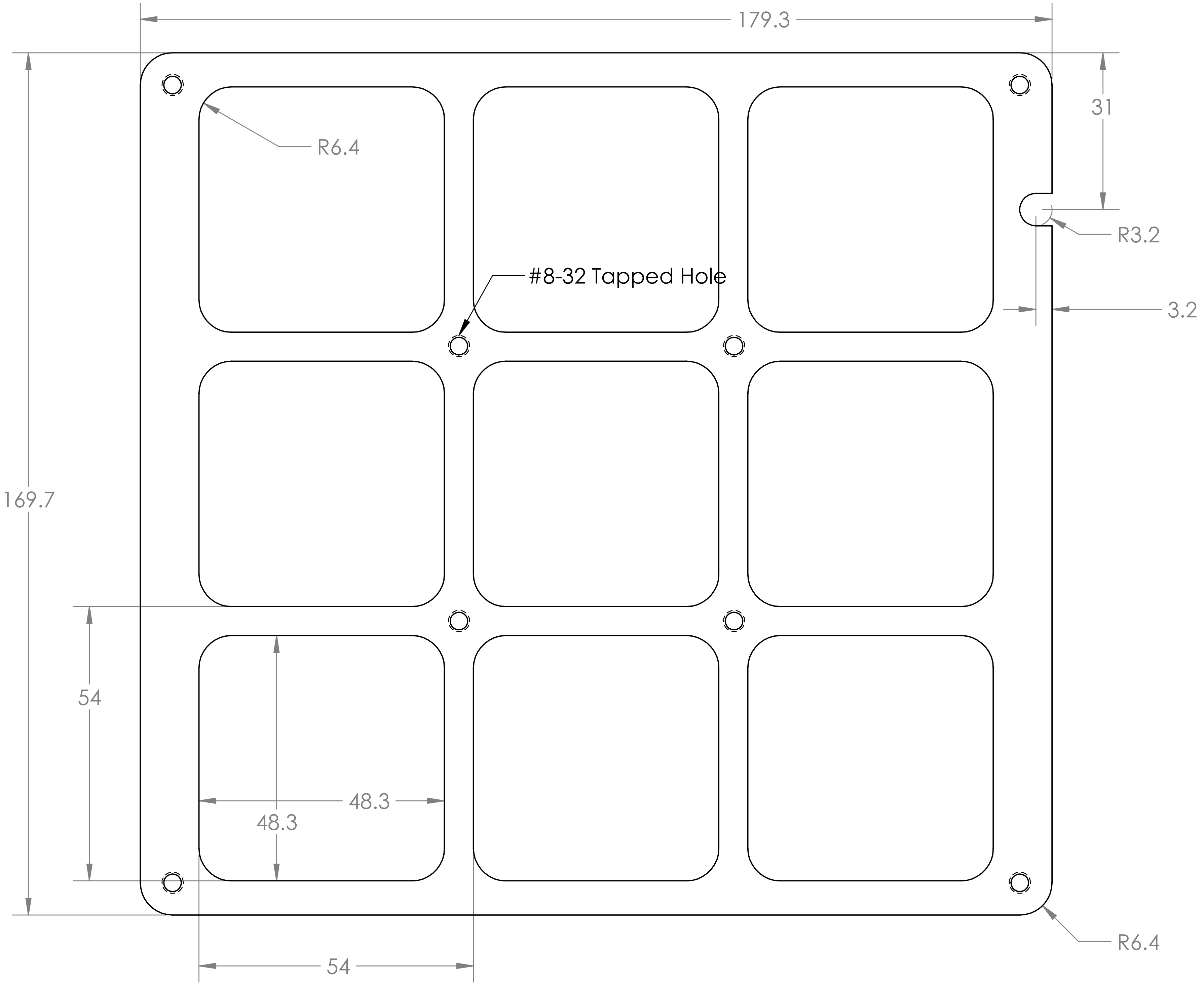

2-INCH POT

UNLESS OTHERWISE SPECIFIED:
DIMENSIONS ARE IN INCHES
TOLERANCES:
FRACTIONAL ± 1/64
ANGULAR: MACH ± .1° BEND ±
TWO PLACE DECIMAL ± .02
THREE PLACE DECIMAL ± .002

MATERIAL
3MM PVC
FINISH

DO NOT SCALE DRAWING

DRAWN	SAVAGE
DATE	7/23/19
SCALE	1:1
SHEET	
JOB #	430B/19

COMMENTS:

FILEPATH:

WSU TECHNICAL SERVICES

TITLE:
PLANT PHOTO CRATE

SIZE	DWG. NO.	REV
B		
