# Supplementary material for: Fluctuating light experiments and semi-automated plant phenotyping enabled by self-built growth racks and simple upgrades to the IMAGING-PAM
Source: Plant Methods. 2019 Dec 23;15:156. doi: 10.1186/s13007-019-0546-1 (PMC6927185; doi:10.1186/s13007-019-0546-1)
Supplement: Supplementary file 4 — Additional file 4. Post-processing quality control output generated by the ImagingPAMProcessing toolkit. The proof-of-concept dataset from this was used for this output example. [file 13007_2019_546_MOESM4_ESM.html]

Post-Processing QC


# Post-Processing QC

#### Dominik Schneider

#### Sep 20, 2019

# 1 Read data from image analysis

# 2 Check image results

During image processing the detected objects are checked for:

1. whether the plant is completely in frame (top row)
2. whether the plants are unique (bottom row)

# 3 Subset Valid data

For subsequent plots we will remove invalid datapoints based on the quality checks above. These are saved as the “level1” dataset.

# 4 Plant Area

# 5 YII

## 5.1 Steady-state YII

# 6 NPQ

## 6.1 Steady-state NPQ

# 7 Treatment Effects

## 7.1 How does each genotype respond to the treatment compared to control (steady light) conditions?

### 7.1.1 Plant Area

### 7.1.2 YII

#### 7.1.2.1 Steady-state YII

### 7.1.3 NPQ

#### 7.1.3.1 Steady-state NPQ

## 7.2 How does each mutant respond to the treatment compared to WT?

### 7.2.1 Plant Area

### 7.2.2 YII

#### 7.2.2.1 Steady-state YII

### 7.2.3 NPQ

#### 7.2.3.1 Steady-state NPQ

# 8 Compilation for Manuscript
