# Supplementary figures and images for: Fluctuating light experiments and semi-automated plant phenotyping enabled by self-built growth racks and simple upgrades to the IMAGING-PAM
Source: Plant Methods. 2019 Dec 23;15:156. doi: 10.1186/s13007-019-0546-1 (PMC6927185; doi:10.1186/s13007-019-0546-1)

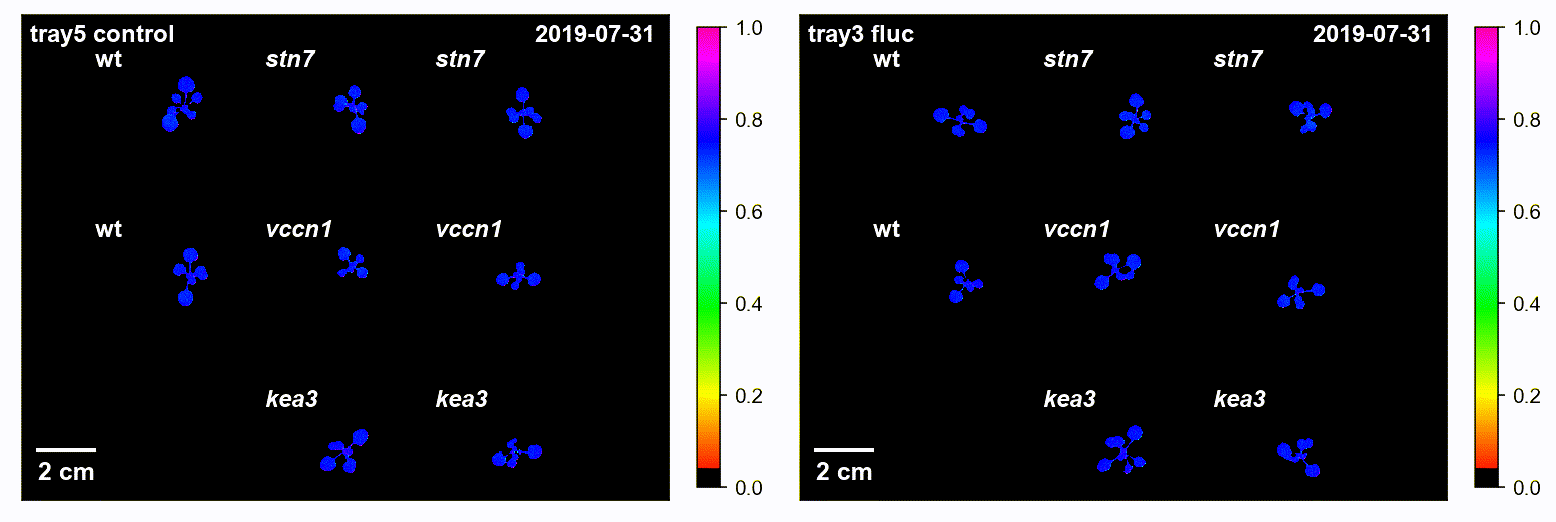

Supplement: Supplementary file 5 — Additional file 5. Time-lapse movie for Fv/Fm generated from the proof-of-concept dataset. [file 13007_2019_546_MOESM5_ESM.gif]

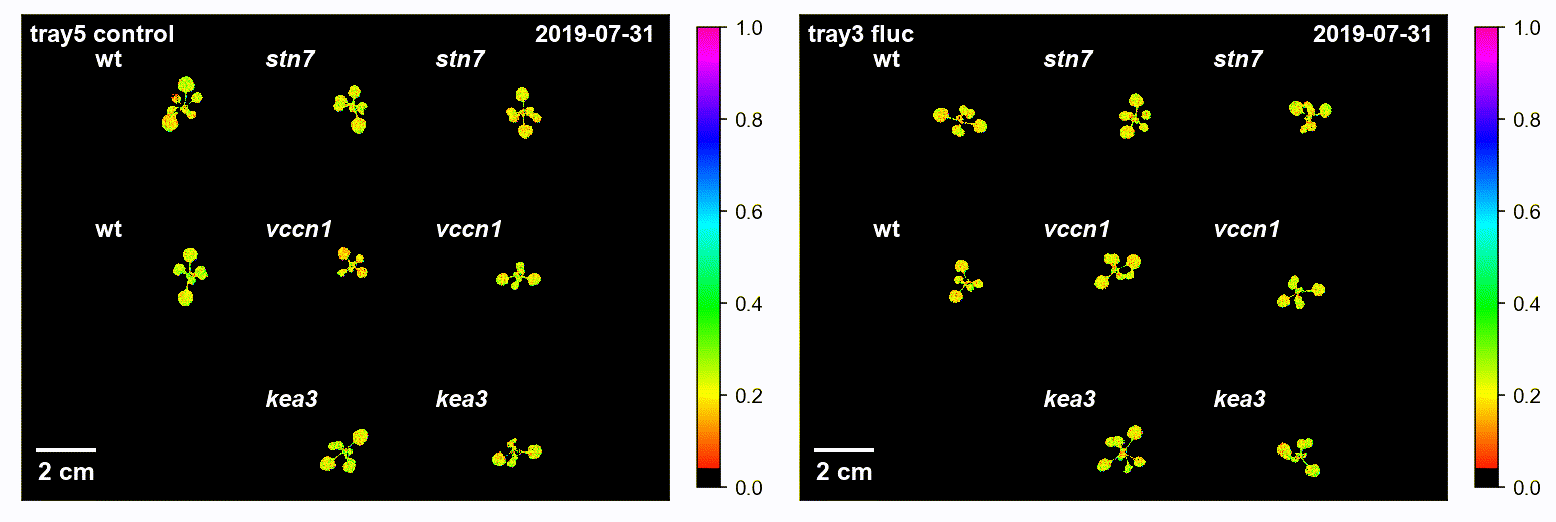

Supplement: Supplementary file 6 — Additional file 6. Time-lapse movie for steady-state YII generated from the proof-of-concept dataset. [file 13007_2019_546_MOESM6_ESM.gif]

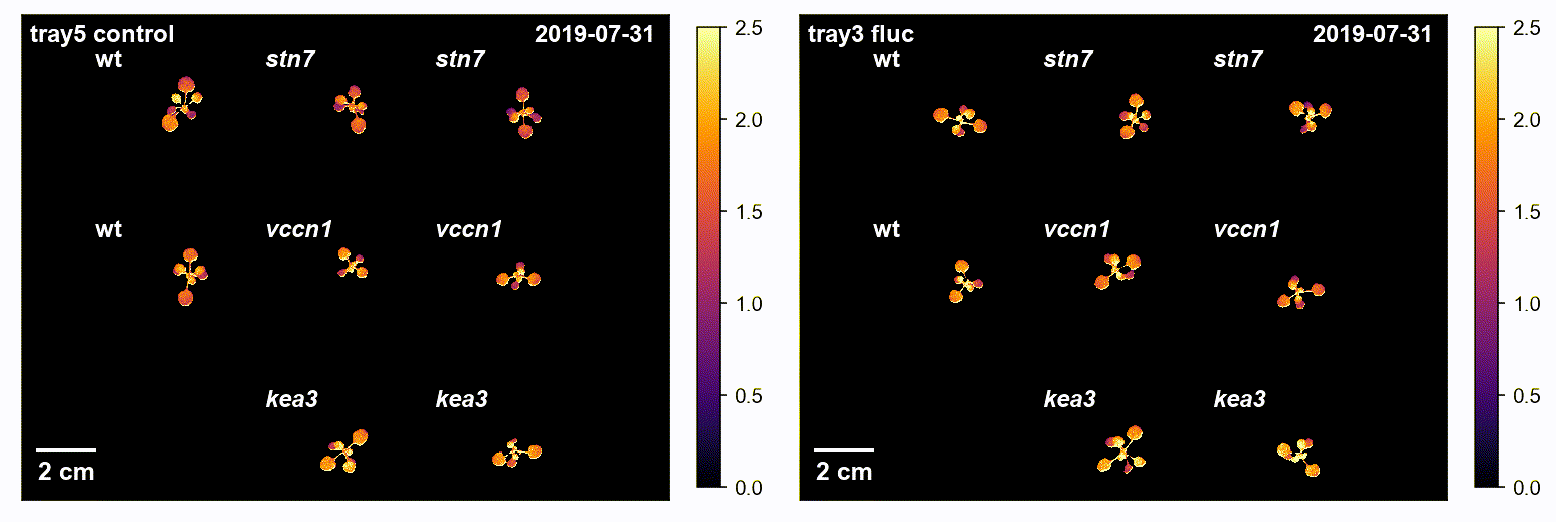

Supplement: Supplementary file 7 — Additional file 7. Time-lapse movie for steady-state NPQ generated from the proof-of-concept dataset. [file 13007_2019_546_MOESM7_ESM.gif]
